# Long-Term Stimulant Treatment Affects Brain Dopamine Transporter Level in Patients with Attention Deficit Hyperactive Disorder

**DOI:** 10.1371/journal.pone.0063023

**Published:** 2013-05-15

**Authors:** Gene-Jack Wang, Nora D. Volkow, Timothy Wigal, Scott H. Kollins, Jeffrey H. Newcorn, Frank Telang, Jean Logan, Millard Jayne, Christopher T. Wong, Hao Han, Joanna S. Fowler, Wei Zhu, James M. Swanson

**Affiliations:** 1 Department of Radiology, Stony Brook University, Stony Brook, New York, United States of America; 2 Bioscience Department, Brookhaven National Laboratory, Upton, New York, United States of America; 3 Department of Psychiatry, Mount Sinai School of Medicine, New York, New York, United States of America; 4 National Institute on Drug Abuse, Bethesda, Maryland, United States of America; 5 Neuroimaging Lab, National Institute on Alcohol Abuse and Alcoholism Intramural Research Program, Upton, New York, United States of America; 6 Department of Pediatrics, University of California Irvine, Irvine, California, United States of America; 7 Department of Psychiatry, Duke University, Durham, North Carolina, United States of America; 8 Department of Mathematics and Applied Sciences, Stony Brook University, Stony Brook, New York, United States of America; Centre national de la recherche scientifique, France

## Abstract

**Objective:**

Brain dopamine dysfunction in attention deficit/hyperactivity disorder (ADHD) could explain why stimulant medications, which increase dopamine signaling, are therapeutically beneficial. However while the acute increases in dopamine induced by stimulant medications have been associated with symptom improvement in ADHD the chronic effects have not been investigated.

**Method:**

We used positron emission tomography and [^11^C]cocaine (dopamine transporter radioligand) to measure dopamine transporter availability in the brains of 18 never-medicated adult ADHD subjects prior to and after 12 months of treatment with methylphenidate and in 11 controls who were also scanned twice at 12 months interval but without stimulant medication. Dopamine transporter availability was quantified as non-displaceable binding potential using a kinetic model for reversible ligands.

**Results:**

Twelve months of methylphenidate treatment increased striatal dopamine transporter availability in ADHD (caudate, putamen and ventral striatum: +24%, p<0.01); whereas there were no changes in control subjects retested at 12-month interval. Comparisons between controls and ADHD participants revealed no significant difference in dopamine transporter availability prior to treatment but showed higher dopamine transporter availability in ADHD participants than control after long-term treatment (caudate: p<0.007; putamen: p<0.005).

**Conclusion:**

Upregulation of dopamine transporter availability during long-term treatment with methylphenidate may decrease treatment efficacy and exacerbate symptoms while not under the effects of the medication. Our findings also suggest that the discrepancies in the literature regarding dopamine transporter availability in ADHD participants (some studies reporting increases, other no changes and other decreases) may reflect, in part, differences in treatment histories.

## Introduction

Attention deficit/hyperactivity disorder (ADHD) is considered to be the most prevalent psychiatric disorder of childhood and its increasingly being recognized in adults too. The disorder is often chronic, with prominent symptoms (inattention, hyperactivity/impulsivity and motivation deficits) and impairment frequently continuing into adulthood. Deficits in dopamine (DA) neurotransmission have been associated with the disorder [1]. In adults, as with youth, stimulant medications (methylphenidate (MPH) or amphetamine), which enhance DA signaling have been used for decades as a first line treatment option [2]. MPH acutely enhances DA signaling by blocking the dopamine transporter (DAT), which is the main mechanism by which DA signals are terminated [1]. However, while the acute DA enhancing effects of MPH have been associated with symptom improvement in children [3] and adults [4] with ADHD very little work has been done to evaluate its chronic effects in brain DA signaling. Inasmuch as DAT appear to adapt to the levels of synaptic DA (downregulating when DA is low and upregulating when DA is high), we hypothesized that chronic MPH treatment would result in upregulation of DAT.

To test this hypothesis, we conducted a Positron Emission Tomography (PET) study using [^11^C]cocaine in 18 stimulant-naïve adult patients with ADHD whom we scanned before and after 1 year of clinical treatment with MPH. The retest PET scan was conducted 24 hours after the last clinical MPH dose to ensure that the acute occupancy of DAT would have dissipated. In parallel we also measured DAT availability in 11 healthy controls at baseline and 12 months later with no intervening medication treatment. We hypothesized that long-term MPH treatment would upregulate DAT availability in the brain in response to stimulant-induced elevations in synaptic DA whereas there would be no changes in DAT availability in controls tested at a 12-month interval.

## Methods

### Subjects

This study was carried out at Brookhaven National Laboratory (BNL) and approved by the local Institutional Review Board (IRB) of record: Committee on Research Involving Human Subjects (CORIHS) of Stony Brook University; Study #: IRBnet: 88634; CORIHS ID: 20055906; BNL IRB#390) under CORIHS' federal wide assurance (FWA) #00000125 and BNL's FWA #00000149; Mount Sinai School of Medicine's (MSSM) IRB #02-0123 under FWA #00005686; University of California at Irvine's (UCI) IRB # 2005-4659 under FWA #00004071; and Duke University IRB #8316-06-3RO under FWA #00009025. Written informed consent was obtained from all participants who came to BNL for imaging studies as well as from their referral institutions after the nature of the experiment was fully explained. A Certificate of Confidentiality (COC) #MH-04-170 has been obtained from the funding institution, the National Institute of Mental Health, to protect subject confidentiality from disclosure. The COC covers subjects at all institutions. Full characteristics of the subjects have been described previously [5], so only a summary will be presented here. We completed assessments in 18 never medicated adult ADHD subjects (6M, 12F) and 11 adult healthy controls (9M, 2F) matched for age, socioeconomic status and education (Table 1). ADHD subjects were recruited from a variety of sources, including clinical referrals to the ADHD programs at MSSM, Duke University and UCI. At least two clinicians interviewed the patients to ensure that they met DSM-IV diagnostic criteria for ADHD, as evidenced by the presence of at least 6 of 9 inattention symptoms (with or without 6 of 9 hyperactive/impulsive symptoms) as ascertained with a semi-structured interview using DSM-IV criteria. In addition, evidence was required from each subject's history that some symptoms of ADHD were present in childhood (before age seven) even when the diagnosis was not made until adulthood. Subjects were excluded if they had a prior history of more than three months of medication treatment for ADHD and excluded if this short treatment occurred within the 6 months prior to the study. They were also excluded it they had a present or past history of substance abuse or addiction (except nicotine). Smoking status was assessed with self-report and in our final sample only one of the controls and one of the ADHD subjects were smokers. Exclusion criteria also included present or past history of psychiatric disease (axis I or II diagnosis other than ADHD), or neurological disease, medical conditions that may alter cerebral function (i.e. cardiovascular, endocrinological, oncological or autoimmune diseases), current use of prescribed or over the counter medications, and/or head trauma with loss of consciousness of more than 30 minutes. All subjects had Hamilton Anxiety [6] and Hamilton Depression [7] scores <19. Control subjects were recruited from advertisements in the local newspapers; exclusion criteria other than allowance for ADHD were the same as for ADHD subjects. Urine drug screens were obtained on all subjects the day of the PET scan to check for psychoactive drug use. Written informed consent was obtained after complete description of the study to the subjects.

**Table 1:** Clinical characteristics of control subjects and ADHD subjects.

	ADHD	Control	*P* Value
Sex			
Women	12	2	
Men	6	9	
Age, mean (SD), years	30.9 (9)	33.2 (6)	NS
Ethnic (A, B, C, H, M)	2, 1, 11, 1, 3	0, 1, 9, 0, 1	
Body mass index	25 (6)	25 (3)	NS
Education, mean (SD), year	15.9 (2.7)	15.3 (2.2)	NS

*Ethnic (A: Asian, B: African American, C: Caucasian American, H: Hispanic American, M: More than one race).

*NS: not significant.

### Clinical Scales

We measured clinical symptoms using the Conners Adult Attention Rating Scale (CAARS) long version [8], which provides self-assessment of ADHD symptoms on a 0 (very minimal) to 3 (very much) point scale and with the Strengths & Weaknesses of ADHD Symptoms & Normal-Behaviors (SWAN) [9].

### Study Design

For the 18 ADHD patients, we provided clinical treatment with MPH for 1 year, and then conducted another PET study with [^11^C]cocaine evaluation of DAT availability in these 18 patients as well as 11 of the controls (without medication). The mean total daily dose used of MPH was in the vicinity of 1 mg/kg or its equivalent in long acting dose form. Progress of treatment was followed sequentially using the CAARS and the Swanson Nolan and Pelham ADHD scale [10]. We also used the SWAN to monitor the improvement of behavior. Subjects were rated using the clinical global impressions scale (CGI)- severity and the CGI- improvement at each medication visit. Subjects were titrated in open fashion during weekly medication visits until they reached optimal response and dose was stabilized. Following that, visits were monthly. The follow up PET imaging was conducted 24 hours after the last clinical dose of MPH to ensure that the acute occupancy of DAT would have dissipated and the estimated density of DAT would be uncontaminated by the pharmacologic effect of MPH at its primary site of action. Plasma MP concentration were measured prior to the PET scan to ensure that detectable levels were not present, which was the case for all of the ADHD participants.

### PET Imaging

PET studies were conducted with a Siemens HR+ tomograph (resolution 4.5×4.5×4.5 mm full width half-maximum) in 3D mode. Dynamic scans were started immediately after injection of 4–10 mCi of [^11^C]cocaine (specific activity 0.5–1.5 Ci/µM at end of bombardment). Dynamic scans were obtained for a total of 60 minutes as previously described [11]. Arterial blood was obtained throughout the procedure to measure the concentration of unchanged [^11^C]cocaine in plasma for quantification of DAT availability [12].

### Data Analysis

Regions of interest analysis in striatum (caudate, putamen, ventral striatum) and in cerebellum were drawn directly on an averaged emission image (summation of images obtained between 10 to 60 minutes for [^11^C]cocaine) [11]. Regions of interest for striatum were obtained bilaterally from the planes where they were best identified (2 slices). Right and left cerebellar (2 slices) regions were obtained in the two planes 1.0 and 1.7 cm above the canthomeatal line. These regions were then projected into the dynamic images to generate time activity curves for striatum and cerebellum. Average values for the striatal and cerebellar regions were computed from the different slices where the regions were obtained. The time activity curves for tissue concentration along with the time activity curves for unchanged tracer in plasma were used to calculate the distribution volume (ml/gm) and the blood to tissue transport constant (K_1_) in striatum and cerebellum using a graphical analyses technique for reversible systems (Logan Plots) [13]. The measure of binding potential (BP_ND_), obtained as the ratio of the distribution volume in striatum to that in cerebellum minus 1, was used to quantify the DAT availability, that is the number of transporters that are free to bind to the radiotracer. These measurements are insensitive to changes in body weight.

### Statistical Analysis

Differences in DAT availability (BP_ND_) for each striatal regions (caudate, putamen, ventral striatum) were evaluated using a repeated (baseline vs. follow-up scan) ANOVA for the comparison between repeated scans within the control or the ADHD group. One between subject factor (group) and one between subject factor (baseline vs. follow-up scan) ANOVA was used for the group comparison (ADHD vs. controls) on changes in DAT (baseline vs. follow-up scan). Differences between the baseline and the follow-up scan are expressed as percent change in BP_ND_ from baseline. The significance level was set at *p*<0.017 after Bonferroni correction (3 regions).

## Results

The ADHD subjects and control subjects are similar in age and socioeconomic background (Table 1). Prior to MPH treatment, each of the following CAARS subscale scores was higher for ADHD subjects than controls: A. Inattention/memory problems; B. Hyperactivity/Restlessness; C. Impulsivity/Emotional liability; D. problems with self concept; E. DSM-IV Inattentive symptoms; E. DSM-IV Hyperactive-impulsive symptoms, G. DSM-IV ADHD total symptom; H. ADHD index. The scores on inattention from the SWAN scale were higher for ADHD participants than for controls (Table 2). During treatment, all the CAARS subscales and the SWAN scores obtained while on medication within the week prior to the PET scan were significantly improved for the ADHD subjects (Table 1). Moreover, except for impulsivity (p<0.02) and inattention (p<0.04), most of the scores for the ADHD subjects while on medication were not significantly different from that of controls.

**Table 2:** Attention rating scales of control subjects in baseline (BL) and in 12 months follow-up (FU) and ADHD subjects prior to (BL) and after 12 months oral methylphenidate treatment (FU).

	ADHD(n = 18)		*P* Value	Control(n = 11)	*P* Value	*P* Value
	BL	FU	BL vs. FU	BL	ADHD-BL vs. Control	ADHD-FU vs. Control
CARRS mean (SD)						
Inattention	26 (6)	11 (8)	0.001	6 (5)	0.001	NS
Hyperactive/restless	23 (9)	8 (7)	0.001	7 (4)	0.001	NS
Impulsivity	20 (7)	8 (7)	0.001	4 (3)	0.001	0.02
Self-concept	9 (4)	4 (4)	0.001	4 (3)	0.001	NS
DSM-IV Inattentive	20 (4)	7 (5)	0.001	4 (4)	0.001	0.04
DSM-IV Hyperactive	16 (5)	6 (4)	0.001	4 (4)	0.001	NS
Total symptoms	36 (6)	13 (8)	0.001	6 (7)	0.001	NS
ADHD index	21 (4)	9 (7)	0.001	4 (4)	0.001	NS
SWAN mean (SD)						
Inattentive	2.2 (0.9)	0.6 (0.5)	0.001	0.5 (0.5)	0.001	NS
Hyperactive	0.6 (0.9)	0.2 (0.4)	0.05	0.3 (0.7)	NS	NS

*CAARS: Conners Adult Attention Rating Scale.

*SWAN: Strengths & Weaknesses of ADHD Symptoms & Normal-Behaviors.

*NS: not significant.

**Figure 1:**
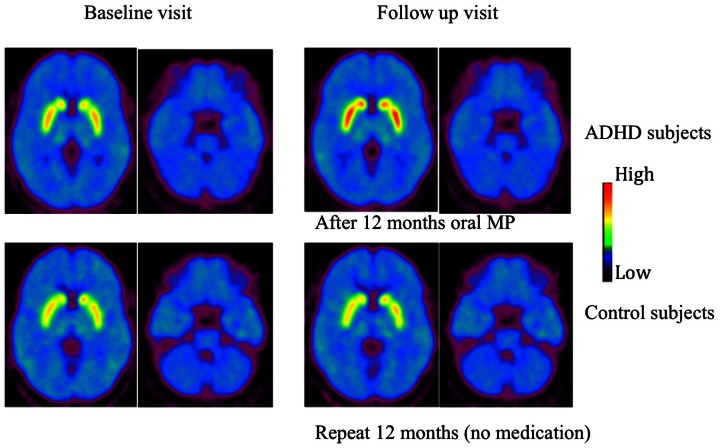
Averaged dopamine transporter availability images. Averaged dopamine transporter availability images of ADHD (n = 18) and control (n = 11) subjects prior to and after 12 months oral MP treatment as well as baseline and 12 follow up scans of control subjects. The images are scaled with respect to the maximum value (distribution volume ratio) obtained on the ADHD subjects at follow up visit and presented using the rainbow scale. Red represents the highest value and dark violet represents the lowest value.

Table 3 shows the measures of DAT availability obtained prior to and after 1 year of MPH for the ADHD participants and for the controls for the baseline and follow-up measures. The repeated measures ANOVA showed that DAT availability in caudate and putamen did not differ between scans (baseline vs. follow-up) neither in the controls nor in the ADHD participants. In contrast the repeated measures differed in the ventral striatum in the ADHD participants was on average 24% higher than that at the baseline (the standard error of this relative percentage change is 32%, *p*<0.01) but the difference was not significant in controls (Figure 1).

**Table 3:** Measures [mean (SD)] of dopamine transporter availability (BP_ND_) of control subjects at baseline (BL) and 12 months follow-up (FU) and ADHD subjects prior to (BL) and after 12 months oral methylphenidate treatment (FU).

	ADHD subjects		*P* Value	Control subjects		*P* Value
	BL	FU	BL vs. FU	BL	FU	BL vs. FU
Caudate	0.8 (0.11)	0.90 (0.2)	NS	0.76 (0.09)	0.71 (0.1)	NS
Putamen	0.95 (0.11)	1.05 (0.19)	NS	0.88 (0.08)	0.88 (0.1)	NS
Ventral Striatum	0.79 (0.15)	0.96 (0.2)	0.01	0.80 (0.12)	0.89 (0.12)	NS

Comparison between BL and FU scans.

The comparison of DAT availability between groups (ADHD vs. Controls) for the baseline scan did not differ (Table 4). It was significantly higher at the 1-year follow-up scan in the caudate (*p*<0.007) and the putamen (*p*<0.005) of the ADHD participants than in the controls. There were no differences at follow up on DAT availability between the groups in the ventral striatum.

**Table 4:** Measures [mean (SD)] of dopamine transporter availability (BP_ND_) of control subjects at baseline (BL) and 12 months follow-up (FU) and ADHD subjects prior to (BL) and after 12 months oral methylphenidate treatment (FU).

	FU scans		*P* Value	BL scans		*P* Value
	ADHD	Control	ADHD vs. Controls	ADHD	Control	ADHD vs. Controls
Caudate	0.90 (0.2)	0.71 (0.1)	0.007	0.8 (0.11)	0.76 (0.09)	NS
Putamen	1.05 (0.19)	0.88 (0.1)	0.005	0.95 (0.11)	0.88 (0.08)	NS
Ventral Striatum	0.96 (0.2)	0.89 (0.12)	NS	0.79 (0.15)	0.80 (0.12)	NS

Comparison of dopamine transporter availability (BP_ND_) between groups (ADHD vs. Controls) for the BL scans and for the FU scans.

## Discussion

This study shows that long-term treatment with MPH up-regulated DAT availability in the ventral striatum, providing the first evidence of DAT neuroplasticity after long-term treatment with a clinically relevant dose of MPH in the human brain. DAT is responsible for recycling DA from the extracellular space into the pre-synaptic terminal [14]. The DAT levels in the membrane are regulated by the concentration of extracellular DA; DAT levels decrease when extracellular DA is low and increase when extracellular DA is high [15]. Repeated administration of a variety of stimulant drugs (e.g., cocaine, amphetamine) has been shown to change DAT expression in preclinical models. These studies show different results for stimulant drugs that are DAT blockers, such as cocaine, from those of stimulant drugs that are DA releasers, such as methamphetamine and amphetamine. Cocaine, which like MPH blocks DAT, temporarily increases the expression of DAT after chronic administration [16]. Indeed humans, postmortem and imaging studies have shown increased DAT (20–50%) in the striatum of chronic cocaine abusers when compared with controls [17], [18]. These increases are positively correlated with the severity of cocaine use and can recover with detoxification. This is consistent with an adaptation response to compensate for chronic increases in extracellular DA secondary to repeated cocaine intoxication.

Similarly, subchronic MPH treatment results in an attenuation of DA release in rodents, which was ascribed to either an upregulation of DAT or enhanced autoreceptor sensitivity [19]. In ADHD adults we also recently showed that long-term treatment with clinical doses of MPH resulted in an attenuation of MPH induced DA increases in the striatum [20]. Similar to treatment with other DAT blockers the increased expression of DAT in the striatum after long term MPH treatment in this study might reflect an accelerated clearance of synaptic DA in response to chronic DA enhancement from long-term exposure to MPH [14]. In this study the clinical measures at follow-up were obtained while subjects were under the influence of the medication (MPH), which explains the significant improvement in all of the clinical symptoms. However it would have been desirable to test them also when they were not under the effects of MPH (i.e. in the morning prior to medication intake) to assess if the upregulation of DAT after chronic MPH was associated with impaired performance.

Few studies have investigated the behavioral consequences of long-term exposure to MPH and the extent to which chronic exposure results in tolerance is still a matter of debate. Indeed, studies on the chronic effects of MPH have reported conflicting results with some documenting sensitization to the locomotor effects of MPH [21], others tolerance [22], and others no changes [23]. The reasons for these discrepancies are likely to reflect differences in doses, conditions of drug administration and age of the animals. The findings on the effects of chronic MPH (using doses that are therapeutically relevant), on the rewarding effects of drugs of abuse are also not consistent. Whereas one study reported that MPH pretreatment in preadolescence or in adulthood decreased the rewarding effects of cocaine (as assessed by conditioned place preference) later in life [24], two others [25], [26] reported that chronic MPH treatment in adolescence or in adulthood enhanced cocaine's reinforcing effects (as assessed by cocaine self-administration and the latency for acquisition of self-administration). These behavioral changes are likely to reflect in part changes in brain DA activity since DA is involved both in locomotor activity as well as the rewarding effects of cocaine. In this study, even though the ADHD subjects did not show more hyperactivity as compared to the controls prior to MPH treatment, the SWAN scores for the hyperactivity/impulsivity dimension in the ADHD subjects were significantly reduced after long-term MPH treatment.

We hypothesize that the increased DAT availability is a compensation for the pharmacologic occupancy of DAT (estimated to be greater than 50%) [27] and the increased elevations in synaptic DA. The results of this prospective treatment study and theory of DAT plasticity suggest that some of the discrepancies in the literature regarding the levels of DAT in ADHD may reflect treatment histories. Note also that in some instances the results are confounded by measuring DAT while the pharmacological effects of MPH are still present [28], which would result in lower measures of DAT availability secondary to DAT occupancy by MPH. Thus we postulate that decreased synaptic levels of DA might drive the changes in DAT levels reported in ADHD (which vary to maintain equilibrium of synaptic DA levels in brain).

### Study Limitations

1. This study was designed as an open MPH treatment protocol. Ideally it would have been desirable to have a have a “double-blind, drug-placebo randomized” design, which would have allowed us to assess the changes in DAT measures in ADHD subjects who are not receiving medication. 2. It would have also been ideal to have a control group that also received 12 months of medication to assess if adaptation changes differ between the controls and the ADHD subjects. However, these other designs raise ethical issues, which make them inappropriate. The current design was constructed to address if there are changes in DAT with long-term treatment but did not evaluate the duration of the DAT increases once medications was discontinued. 3. Several participants were female and in them the DAT measures may be confounded by the time of the menstrual cycle at which the studies were performed, which we did not controls nor did we measure gonadal hormones. 4. Since we did not obtain clinical measures for the follow up scans while subjects were not under the acute effects of MPH it is not possible for us to test whether the increases in DAT observed with chronic treatment were associated with worsening of inattention.

## Conclusion

Here we report an upregulation of DAT secondary to long-term treatment with stimulant medication, which could result in further decreases in dopaminergic signaling when the individual with ADHD is not medicated (i.e. over weekend holidays). To the extent that reduced DA release in ADHD is associated with inattention [29], this could result in more severe inattention and the need for higher doses of medication. Though there is limited literature on loss of efficacy of stimulant medication with long-term treatment this is an area that merits further investigation. Studies are necessary to test if DAT down-regulate after MPH discontinuation and the time necessary for their recovery.
